# Mixed Infection With Aspergillosis and Actinomycosis in the Maxillary Sinus: A Case Report

**DOI:** 10.7759/cureus.91758

**Published:** 2025-09-07

**Authors:** Reiji Kokubo, Ryunosuke Umehara, Yoshiaki Katada, Natsuhiko Shirota, Shinji Sugahara, Kazuhiro Saito, Ryo Akai, Yukio Morishita

**Affiliations:** 1 Department of Radiology, Tokyo Medical University Ibaraki Medical Center, Ibaraki, JPN; 2 Department of Radiology, Tokyo Medical University, Tokyo, JPN; 3 Department of Otorhinolaryngology, Tokyo Medical University Ibaraki Medical Center, Ibaraki, JPN; 4 Department of Diagnostic Pathology, Tokyo Medical University Ibaraki Medical Center, Ibaraki, JPN

**Keywords:** actinomycosis, aspergillosis, case report, maxillary sinusitis, mixed infection

## Abstract

This report describes a rare case of aspergillosis and actinomycosis occurring simultaneously in the maxillary sinus. A 74-year-old female patient visited our institution due to a chief complaint of left purulent nasal discharge. Her medical history included rheumatoid arthritis (which was treated with oral methotrexate) and diabetes mellitus. Computed tomography scan revealed soft tissue shadows and high-density areas, which are indicative of fungal balls, in the left maxillary sinus, ethmoid sinus, and frontal sinus. Magnetic resonance imaging showed a significantly low signal on the T2-weighted image that corresponded to the high-density areas observed on the computed tomography scan. Sinus fungal infection was strongly suspected, and type III endoscopic sinus surgery was performed. During the surgery, a large volume of bacterial mass-like materials with a foul odor indicating anaerobic infection was observed after the maxillary sinus was opened. Histopathological examination confirmed Aspergillus hyphae and actinomycete masses (with the Splendore-Hoeppli phenomenon). The patient was diagnosed with mixed infection with aspergillosis and actinomycosis. After the surgery, the patient was treated with penicillin drip and oral amoxicillin, and the patient exhibited a favorable clinical progress. This type of mixed infection is extremely rare. Herein, we report a rare case of mixed infection with aspergillosis and actinomycosis, and a literature review was performed.

## Introduction

Fungal sinus infection, particularly fungus balls (FBs) caused by Aspergillus, is a relatively common disease [1]. However, sinusitis caused by actinomycosis is a rare bacterial infection, and the risk factors are dental procedures, presence of diabetes, and immunosuppressive status [2-4]. Imaging is important for diagnosing these diseases. FB is characterized by high absorption areas (including calcification) on computed tomography (CT) scan and significant low signal intensity on T2-weighted magnetic resonance imaging (MRI) [5]. Actinomycosis is characterized by bone sclerosis of the sinus wall on CT scan [3]. However, it may resemble FB, and MRI findings are considered nonspecific [6]. The treatments for the two conditions also differ. In particular, FB is usually cured by surgical resection alone, and actinomycosis requires long-term antibiotic therapy in addition to surgical treatment [4,6]. Mixed infection with aspergillosis and actinomycosis is extremely rare, and previous reports on its imaging findings and clinical characteristics are limited. Herein, we report a case in which FB was strongly suspected on imaging and multiple risk factors were present. Nevertheless, the condition was ultimately diagnosed as mixed infection with aspergillosis and actinomycosis in the maxillary sinus.

## Case presentation

**Figure 1:**
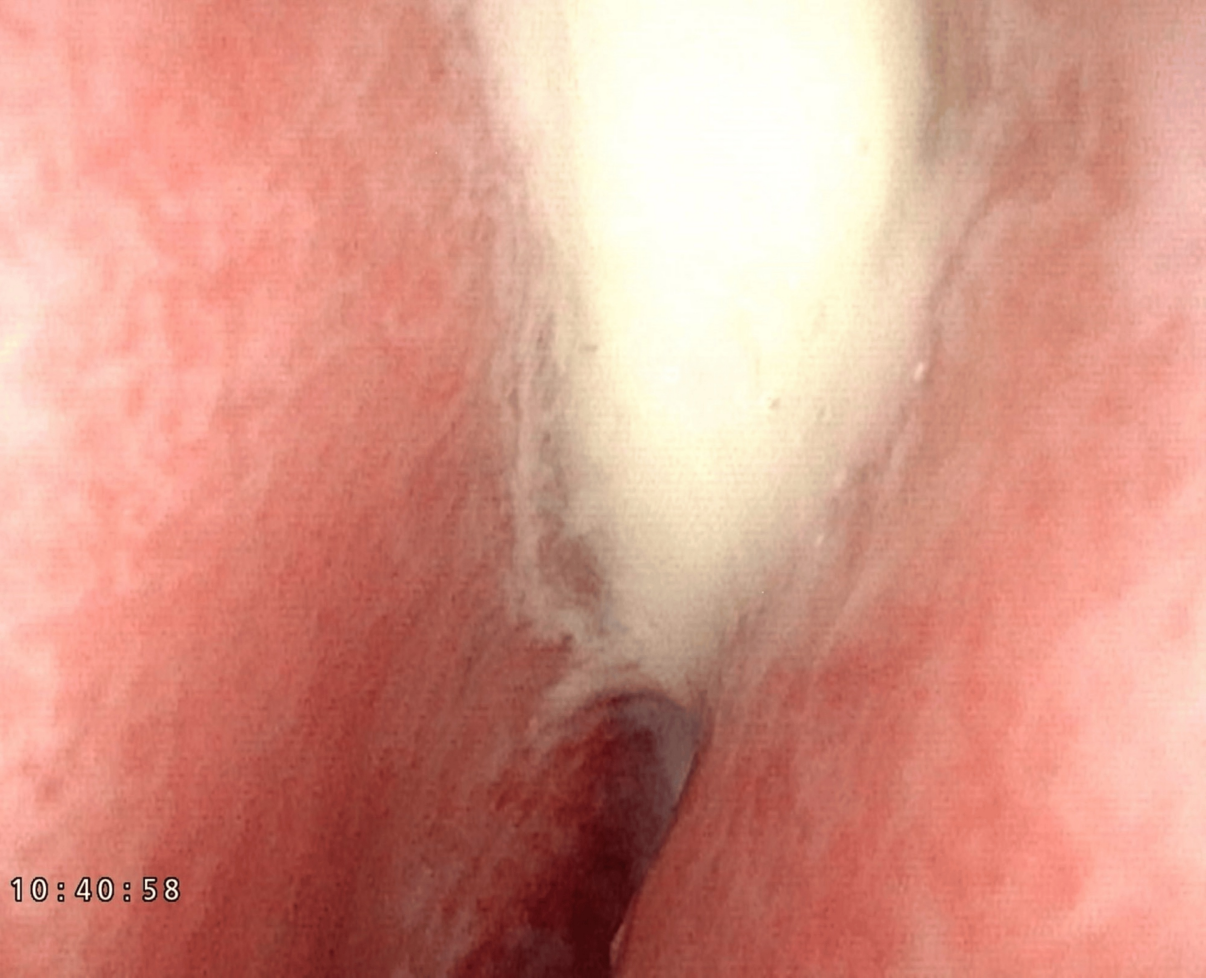
Nasal endoscopy showing the left middle meatus filled with purulent discharge.

**Figure 2:**
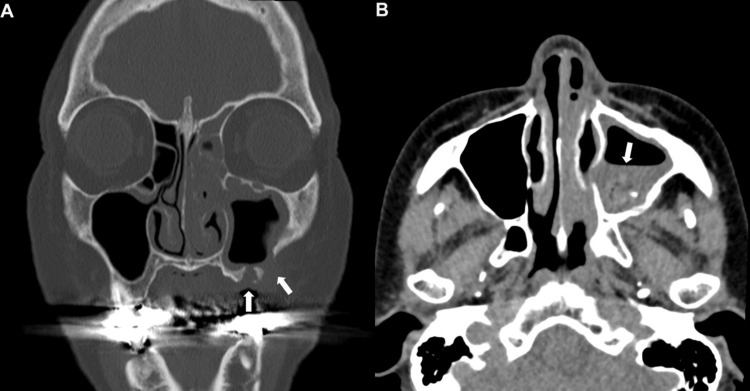
Coronal bone-window CT scan (A) showing mucosal thickening in the left maxillary, ethmoid, and frontal sinuses, with reactive thickening of the left maxillary sinus wall. Changes of post-implant removal and a Caldwell-Luc antrostomy are visible (white arrow), with no bone destruction. Axial soft-tissue window CT scan (B) showing a roundish high-density area with punctate calcifications in the center of the left maxillary sinus (white arrow).

**Figure 3:**
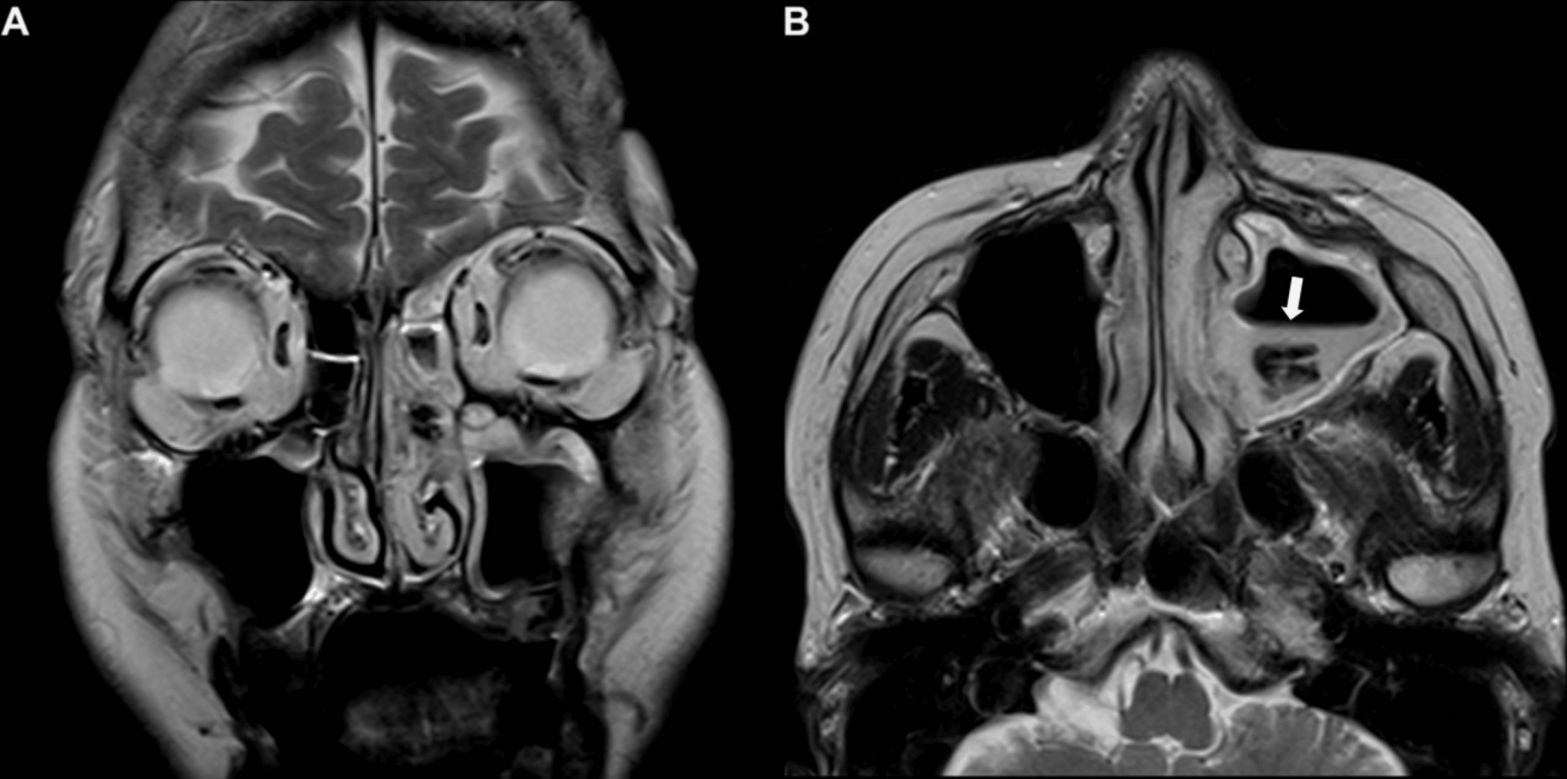
Coronal T2WI (A) showing a high signal intensity along the left maxillary sinus wall, extending to the left ethmoid and frontal sinuses, consistent with mucosal thickening. Axial T2WI (B) showing an air-fluid level (high signal) posteriorly in the maxillary sinus and significant low signal intensity within the sinus corresponding to the high-density area on CT scan (white arrow), which are indicative of a fungus ball.

**Figure 4:**
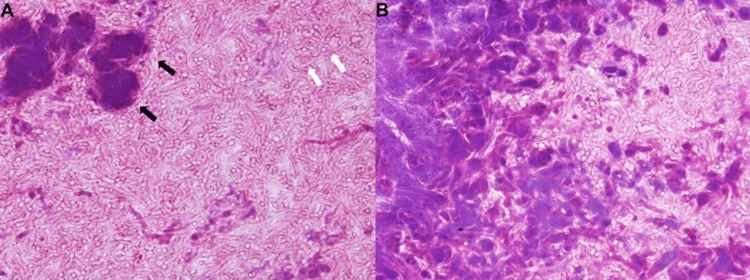
(A) Hematoxylin and eosin stain (×400) showing numerous septate hyphae with acute-angle (~45°) branching (white arrow, Aspergillus) and bacterial colonies (“sulfur granules”) surrounded by eosinophilic radial material consistent with the Splendore–Hoeppli phenomenon (black arrow, Actinomyces). (B) Hematoxylin and eosin stain (×400) showing extensive intermingling of Aspergillus hyphae and Actinomyces colonies.

**Figure 5:**
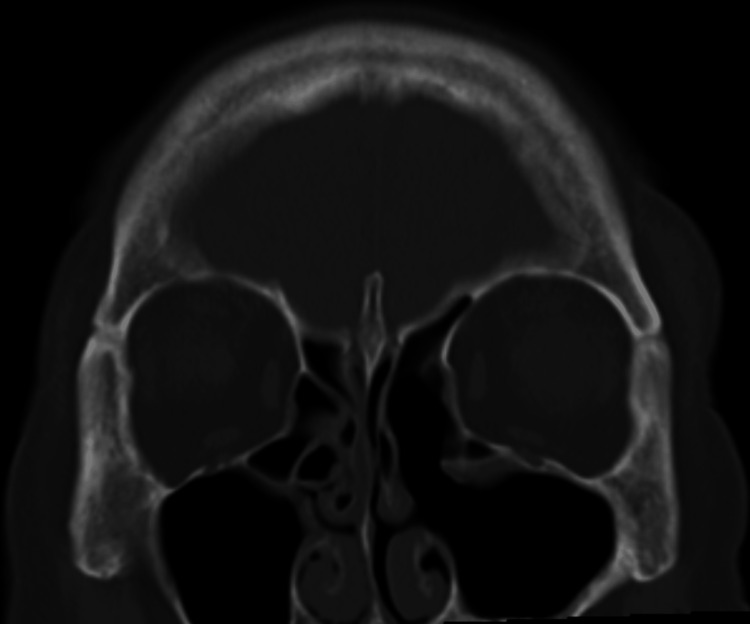
Coronal CT scan of the head performed eight months post-ESS for an unrelated issue showing that the left maxillary and ethmoid sinuses were clear, with no mucosal thickening or fluid retention, and good aeration. ESS: endoscopic sinus surgery

The patient was a 74-year-old woman. She had been experiencing purulent nasal discharge for 1.5 years. Her dentist removed the implants on her upper left teeth 4-7, and three left maxillary gingival incisions were made. However, her symptoms did not improve. She visited the otolaryngology department of our institution. Her medical history included rheumatoid arthritis, diabetes, hypertension, and asthma. She was receiving methotrexate 10 mg/day, linagliptin 5 mg/day, amlodipine besylate 2.5 mg/day, and once-daily inhaled glycopyrronium/indacaterol. The endoscopic findings were a deviated left nasal septum, and the left middle nasal meatus was filled with purulent nasal discharge (Figure 1). No notable findings were found in the right nasal cavity. The blood test results showed a normal white blood cell count (5400/µL) and β-D-glucan level (1.4 pg/mL), and the patient’s hemoglobin A1c level was 6.2%, which was slightly elevated. CT scan showed mucosal thickening and soft tissue shadows in the left maxillary sinus, with some high-density and punctate calcifications at the center. Soft tissue shadows were also observed in the left ethmoid sinus, left frontal sinus, and nasal cavity. The left maxillary sinus wall was thickened reactively, indicating a chronic course. Changes were noted in the left maxillary teeth 4-7 after implant removal. Bone holes were observed after the Caldwell-Luc antrostomy. However, no bone destruction was noted (Figure 2). T2-weighted imaging (T2WI) MRI showed a high signal intensity area along the left maxillary sinus wall, extending from the left ethmoid sinus to the left frontal sinus, which indicated mucosal thickening. A high signal intensity area on T2WI indicating air-fluid level was found on the dorsal side, suggesting fluid retention. The area corresponded to a region exhibiting high absorption on CT scan, and a strong low signal indicating an FB was observed on T2WI (Figure 3). Treatment with clarithromycin 200 mg/day was started. After approximately one month, the antibiotic was changed to lascufloxacin at a dose of 75 mg/day. Antibiotic treatment was ineffective, and the CT scan and MRI findings suggested sinus fungal infection. Thus, left-sided type III endoscopic sinus surgery (ESS) was performed. During surgery, when the maxillary sinus was opened, a large amount of bacterial mass-like material and a foul odor indicative of anaerobic bacterial infection were observed. The anterior and posterior ethmoid sinuses, maxillary sinus, and frontal sinus were opened via the anterior naris, and the accumulated material in the sinuses was removed. In the histopathological examination, hematoxylin and eosin stain revealed numerous septate hyphae of uniform width, with acute-angle (~45°) branching, consistent with Aspergillus, and bacterial colonies (“sulfur granules”), consistent with Actinomyces (Figure 4). Moreover, eosinophilic radial deposits (the Splendore-Hoeppli phenomenon) were observed around them. A large number of both organisms were present. Based on the abovementioned pathological findings, a definite diagnosis of mixed infection with maxillary sinus aspergillosis and actinomycosis was made. No systemic antifungal therapy was administered because the aspergillosis was diagnosed as a non-invasive fungus ball, which is considered curable by complete surgical removal. The actinomycosis component was treated with penicillin (24 million units/day) intravenously for six days from the day of surgery and with oral amoxicillin (1500 mg/day) for approximately one month from the time of discharge. Two months after the surgery, the subjective symptoms improved, and the postoperative course was favorable. Thus, the patient was discharged. Eight months after the surgery, a head CT scan was performed to assess for other diseases. Results showed that the soft tissue shadow in the left paranasal sinus had disappeared and that the paranasal sinus was well-filled (Figure 5). Long-term follow-up was planned to monitor for recurrence.

## Discussion

Aspergillus is a fungus that is widespread in the environment, and it lives in decaying organic matter. In 1885, Schubert reported the first case of aspergillosis of the nasal sinuses [1,7]. Sinus aspergillosis is classified into the invasive and noninvasive types, depending on the presence or absence of mycelium invasion into the mucosal tissue in the pathological tissue. In the noninvasive type, it is classified as aspergilloma (FB) and allergic fungal rhinosinusitis. The most common type is FB, which is a ball of noninvasive fungal hyphae. FB usually occurs in one maxillary or sphenoid sinus, has a female predisposition, and typically develops in late adulthood. Its common risk factors are previous root canal treatment and apical closure [8]. In contrast, actinomycetes are gram-positive filamentous bacteria that are non-acid-fast and anaerobic to microaerophilic. They usually colonize the human oral cavity, urogenital tract, and gastrointestinal tract, and they can cause an infectious disease referred to as actinomycosis [9]. Actinomycosis of the paranasal sinuses was first described by Ponfick in 1882 and was subsequently described, specifically in the maxillary sinus, by Stanton in 1966 [10,11]. The risk factors for actinomycosis include dental surgery, facial fractures, diabetes, immunosuppression, and poor dental hygiene [2-4]. In this case, several risk factors that could have contributed to the mixed infection with sinus fungal infection and actinomycosis were identified. These included a history of methotrexate use for rheumatoid arthritis, immunosuppression due to diabetes, dental implant removal, and post-Caldwell-Luc procedure.

CT scans of the paranasal sinus aspergilloma showed almost complete opacification and microcalcification of the affected sinus, and contrast-enhanced CT scan revealed mucosal thickening around the FB. MRI scans showed a significantly low signal on T2-weighted images [5]. In contrast, CT scan findings of paranasal sinus actinomycosis are characterized by soft tissue shadows in the affected sinus, bone sclerosis of the sinus wall, and calcification, which are similar to those of FB. MRI findings of actinomycosis are nonspecific [3,6]. Imaging findings such as severe infiltration, strong enhancement after contrast administration, and purulent necrosis within the mass are useful in differentiating actinomycosis [12]. In this case, these findings, which are specific to actinomycosis, were not observed, and the imaging findings were nonspecific and consistent with paranasal sinus fungal infection. The presence of Aspergillus and actinomycosis could not be confirmed based on the imaging findings alone.

Surgical resection is the treatment of choice for actinomycosis and FB. Among the types of surgical resection, ESS is preferred because it facilitates complete resection and restoration of ventilation. In cases of FB, surgical removal alone is usually curative, and systemic antifungal therapy is not routinely required, as there is no tissue invasion [13]. In contrast, medical treatment, particularly antibiotics after surgical resection, is recommended for actinomycosis [6]. In this case, ESS alone successfully treated the non-invasive aspergillosis, whereas the actinomycosis was managed with intravenous penicillin (24 million units/day) for six days followed by oral amoxicillin (1500 mg/day) for approximately one month, resulting in clinical improvement. Due to the difference in treatment methods, it is clinically important to accurately diagnose individual conditions or their coexistence. However, it is challenging to make an appropriate diagnosis based solely on imaging findings. Therefore, even if the imaging findings are relatively typical and indicative of fungal infection, if there are risk factors such as an immunosuppressed state and a history of tooth extraction, as in this case, it is important to consider the possibility of actinomycosis or a mixed infection, and it is essential to select the diagnosis and treatment with consideration of the histopathological examination results.

## Conclusions

Herein, we present a rare case of mixed infection with aspergillosis and actinomycosis in the maxillary sinus of a 74-year-old woman with multiple risk factors including rheumatoid arthritis (which was treated with oral methotrexate), diabetes, and a history of dental treatment and maxillary sinus surgery. The imaging findings strongly suggested FB. Nevertheless, a definitive diagnosis of mixed infection with FB and actinomycosis was made based on the histopathological examination results. The patient was cured with ESS and postoperative antibiotic treatment. In cases involving overlapping risk factors, it is important to consider the possibility of a single or mixed infection with actinomycosis and to select an appropriate treatment based on the pathological diagnosis.
